# Plant Available Zinc Is Influenced by Landscape Position in the Amhara Region, Ethiopia

**DOI:** 10.3390/plants10020254

**Published:** 2021-01-28

**Authors:** Mesfin K. Desta, Martin R. Broadley, Steve P. McGrath, Javier Hernandez-Allica, Kirsty L. Hassall, Samuel Gameda, Tilahun Amede, Stephan M. Haefele

**Affiliations:** 1 Sustainable Agriculture Sciences Department, Rothamsted Research, West Common, Harpenden, Hertfordshire AL5 2JQ, UK; steve.mcgrath@rothamsted.ac.uk (S.P.M.); javier.hernandez@rothamsted.ac.uk (J.H.-A.); kirsty.hassall@rothamsted.ac.uk (K.L.H.); stephan.haefele@rothamsted.ac.uk (S.M.H.); 2 Future Food Beacon of Excellence and School of Biosciences, University of Nottingham, Nottingham LE12 5RD, UK; Martin.Broadley@nottingham.ac.uk; 3 International Maize and Wheat Improvement Center (CIMMYT), ILRI Campus P.O. Box 5689, Addis Ababa, Ethiopia; S.Gameda@cgiar.org; 4 International Crops Research Institute for the Semi-Arid Tropics (ICRISAT), ILRI Campus P.O. Box 5689, Addis Ababa, Ethiopia; T.Amede@cgiar.org

**Keywords:** Adsorption, desorption, landscape position, isotherm, plant available Zn

## Abstract

Zinc (Zn) is an important element determining the grain quality of staple food crops and deficient in many Ethiopian soils. However, farming systems are highly variable in Ethiopia due to different soil types and landscape cropping positions. Zinc availability and uptake by plants from soil and fertilizer sources are governed by the retention and release potential of the soil, usually termed as adsorption and desorption, respectively. The aim of this study was to characterize the amount of plant available Zn at different landscape positions. During the 2018/19 cropping season, adsorption-desorption studies were carried out on soil samples collected from on-farm trials conducted at Aba Gerima, Debre Mewi and Markuma in the Amhara Region. In all locations and landscape positions, adsorption and desorption increased with increasing Zn additions. The amount of adsorption and desorption was highly associated with the soil pH, the soil organic carbon concentration and cation exchange capacity, and these factors are linked to landscape positions. The Freundlich isotherm fitted very well to Zn adsorption (r^2^ 0.87–0.99) and desorption (r^2^ 0.92–0.99), while the Langmuir isotherm only fitted to Zn desorption (r^2^ 0.70–0.93). Multiple regression models developed by determining the most influential soil parameters for Zn availability could be used to inform Zn fertilizer management strategies for different locations and landscape positions in this region, and thereby improve plant Zn use efficiency.

## 1. Introduction

Zinc (Zn) is a trace metal essential to all forms of life because of its fundamental role in gene expression, cell development and replication [1]. In plants, it plays a key role in various enzymatic reactions such as the synthesis of auxin, metabolic processes, and oxidation reduction reactions. It also participates in chlorophyll formation and is essential for many enzymes which are vital for nitrogen metabolism, energy transfer and protein synthesis [2]. Zn has been classed as a catalytic, structural, and regulatory ion [3]. It also has a critical effect on cellular homeostasis. Deficiencies of Zn in people are also widespread due to a lack of dietary intake, which is of public health importance [4,5,6].

Zn deficiencies are common on many cultivated soils in Ethiopia. Soil types, texture, pH, soil organic carbon (SOC), available phosphorus (P), total and available copper (Cu) and iron (Fe), exchangeable cations and cation exchange capacity (CEC) are the main contributors to the extent of Zn deficiency [7,8]. Zinc deficiency has been reported on several soil types in Ethiopia, for example, on Nitisols [9], Nitisols, Vertisols, Fluvisols and Cambisols [10], Vertisols [11], and in a review on Vertisols, Cambisols, Fluvisols, Nitisols, Andisols and Alfisols [12]. In addition, Zn deficiencies were also linked to Cambisols, Luvisols and Regosols of the Tigray region [8], and to salt affected soils of Eastern Ethiopia [13]. These deficiencies along with the potentially low Zn concentration in the crops grown on these soils may cause serious impacts on human health [5,6].

Adsorption and desorption of nutrient ions are the primary processes that affect transport of nutrients and contaminants in soils [14]. Adsorption refers to the quantity of a nutrient that is retained on soil exchange surfaces while desorption is the release from these surfaces; both occur in a system in the state of equilibrium. These are usually described through isotherms, showing the amount of adsorbed/desorbed nutrient in the solid phase (soil colloids) as a function of the concentration of that nutrient in the liquid phase (soil solution), determined at equilibrium conditions and a constant temperature. Although various isotherms have been developed, the two most commonly used isotherms are the Langmuir and Freundlich isotherms. The relationships between adsorption-desorption characteristics and soil properties have been extensively studied on metals such as Zn, Cu and others. Amongst soil properties, pH, clay content, cation exchange capacity (CEC), SOC and hydrous oxides exert the most significant influence on the adsorption-desorption reactions of Zn in soils and, thus, regulate the amount of Zn dissolved in soil solution [7,8,15,16,17,18].

Generally, the solubility of Zn in the soil decreases 100-fold for each unit increase in soil pH [19]. This is due to the greater adsorptive capacity of the soil solid surfaces resulting from increased pH-dependent negative charges, the formation of hydrolyzed forms of Zn, chemisorption on calcite and co-precipitation as Fe oxides [7]. Similarly, [7] reported that high pH and electrical conductivity (EC) are responsible for low availability of Zn in soils. For example, Zn concentration of teff (*Eragrostis tef*, (Zucc.) Trotter]) and wheat (*Triticum asetivum*, L.) leaves were significantly and positively correlated with soil Zn and soil organic carbon, respectively while negatively correlated with pH and CEC of soils in the Tigray Region [8].

Ethiopian farming systems and landscape positions are highly variable and hence nutrient mobility in the soil and their effect on plant Zn uptake and grain quality are also likely to vary. Although the application of Zn as a fertilizer proved to enhance the productivity and quality of staple food crops to some extent in Ethiopia [8], this is not always the case. Therefore, it is important to devise a mechanism for stratified nutrient management options for these systems. Improving the grain Zn content of staple food crops can only be achieved through a better understanding of Zn dynamics in these soils. One way to do this is through adsorption-desorption studies.

The aim of this study was, therefore, to better understand the influence of different landscape positions (upslope, midslope, and footslope) and the associated soil properties on the amount of Zn adsorbed and desorbed in typical soils of Ethiopia. The fitness of the most common adsorption-desorption isotherms for these soils was tested, to identify the dominant soil characteristics driving these processes. Multiple regression models were used, which can be used to inform the amount of adsorbed and desorbed Zn and which in turn could be used to help devise stratified Zn fertilizer recommendations and improve crop Zn use efficiency for these systems and landscape positions.

## 2. Materials and Methods

### 2.1. Site Description

**Figure 1:**
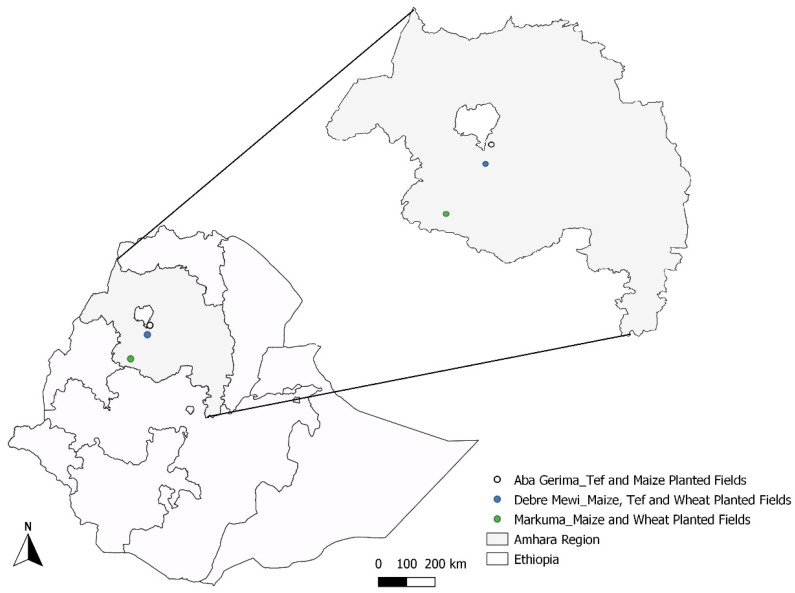
Location of the study sites in the Amhara region, Ethiopia.

The soils used in this study were collected from on-farm trials during the 2018/19 cropping season in the Amhara Region of Ethiopia. Experimental sites were at locations in three districts of the Amhara Region (Bahir Dar Zuriya, Enarj Enawega and Bure Districts), named Aba Gerima, Debre Mewi, and Markuma, respectively (Figure 1). The climate in the region is subtropical with an average annual rainfall of 1022 mm at Aba Gerima, 1240 mm at Debre Mewi and 1450 mm at Markuma and annual minimum and maximum temperatures of 12 and 30 °C respectively [20]. The experimental locations are characterized by hilly landscapes on a plateau at about 1800 to 2200 m ASL. Experimental fields were chosen based on landscape position which in this region has strong effects on soil characteristics (Table 1). Landscape position determines erosion/accumulation of soil particles, causes a notable shift of clay and organic matter concentrations, and of soil colour. The soils at Aba Gerima are highly degraded on the upslope with a clear clay movement to the footslope (Table 2). Few landscape position effects were observed at Markuma which has relatively gentle slopes. The most dominant soil types for all locations were Nitisols but Vertisols were observed in the footslope of Debre Mewi.

The dominant crops grown were: Aba Gerima—tef, maize and finger millet; Debre Mewi—tef, wheat and maize, and Markuma—maize and wheat. At Aba Gerima and going down the slope, there was a shift of crops from the other cereals to finger millet which was sown in high planting density on the footslope. Likewise at Debre Mewi, but the crops are limited to tef and wheat on the footslope with predominantly maize on the upslope, whereas the Markuma sites consistently grow maize and wheat across all landscape positions.

### 2.2. Soil Sample Collection, Preparation and Standard Analysis

Geo-referenced representative soil samples were collected from 60 on-farm experiments in the 2018/19 cropping season. The soil samples were top-soils (0–20 cm depth), combined from 5 sub-samples in each plot, and were collected just before the cropping season. The individual experimental fields were chosen based on landscape position (upslope, midslope, and footslope) and crop grown, i.e., teff (*Eragrostis tef*, (Zucc.) Trotter]), maize (*Zea maize*, L.), wheat (*Triticum asetivum*, L.) or finger millet (*Eleusine coracana* (L.) Gaertn.). These soil samples were used for adsorption-desorption studies. Approximately 100 g of each sample were air dried at ambient temperature (25 °C), ground with mortar and pestle and passed through a 2-mm-sieve.

All samples were subjected to wet chemistry analysis following standard procedures. The soil pH was measured in deionized water, a soil: water ratio of 1:2.5 (10 g of soils with 25 mL of water) and with a temperature compensated two combination pH electrode. Total carbon and nitrogen concentrations were determined by dry combustion [21] using a Leco TruMac CN Combustion Analyser (Leco Corporation, St Joseph, Michigan), and because the pH of all soils was below 6.5, total carbon was assumed to be equivalent to SOC. Available phosphorus (Olsen’s P) was extracted by the sodium bicarbonate method [22]. Phosphorus in the bicarbonate solution was determined using the phospho-molybdenum blue method on the Skalar SANPLUS System (continuous colorimetric flow analysis; Skalar Analytical BV). Total elemental concentrations were measured after an aqua regia extraction [23], followed by inductively coupled plasma optical emission spectrometry (ICP-OES; model, Perkin Elmer Life and Analytical, Shelton, USA). Acid oxalate extractable Fe, Al, Mn and P were determined following extraction with a mixed solution of ammonium oxalate and oxalic acid at a soil: solution ratio of 1:100 [24]. Samples were shaken in the dark (4 h, 20 °C) using a reciprocal shaker, filtered, then acidified and analyzed by ICP-OES. The eCEC determination started with a one-step centrifuge extraction with a 0.0166 M cobalt (III) hexamine chloride solution (Cohex) [Co[NH_3_]_6_]Cl_3_. All exchangeable cations are in the extract while the decrease in Co concentration is a measure of the eCEC, and concentrations were measured by ICP-OES analysis [25]. Soil texture was analyzed using a Laser Scattering Particle Size Distribution Analyser (LA-960, Horbia Ltd., Kyoto, Japan).

### 2.3. Adsorption and Desorption Isotherms

For the adsorption experiments, 0.50 g of soil was equilibrated with 10 mL 0.01 M CaCl_2_ solution containing varying concentrations of ZnSO_4_ × 7H_2_O (0, 2, 5, 10, 15 and 30 mg Zn L^−1^) and shaken end-over-end for 24 h at room temperature. CaCl_2_ was used as the aqueous solvent phase to improve centrifugation and minimize cation exchange [14]. Controls were prepared with only Zn in 0.01 M CaCl_2_ solution (no soil added), for calibration and checking the stability of the test substance in CaCl_2_ solution. A 24-h shaking period was sufficient for complete equilibration of the Zn solutions and the soil in Zn solutions ranging between 1 to 160 mg L^−1^ [26]. The soil and stock solution mixtures were then centrifuged at 3600 rpm for half an hour and the clear supernatant solution was decanted and analyzed for the Zn concentration. This value was set as the Zn equilibrium concentration (Ce), and the difference between the initial stock solution (Co) and the equilibrium solution concentration (Ce) is the adsorbed Zn. To derive desorption isotherms, the original samples were re-suspended with 10 mL of fresh 0.01 M CaCl_2_ stock solution and shaken for 24 h. Again, the mixture was centrifuged, and the supernatant solutions was analyzed for the desorbed Zn concentration (Cde). The amount of Zn adsorbed at equilibrium Qe (mg kg^−1^) was calculated from the following equation [27]:

(1) $$\mathrm{Qe}=\left(\mathrm{Co}-\mathrm{Ce}\right)\frac{V}{W}$$

where Co and Ce (mg L^−1^) are the initial and equilibrium concentrations of Zn in the solution, respectively; Qe (mg kg^−1^) is the amount of adsorbate per unit mass of soil. V is the volume of the solution added (L), and W is the weight of the adsorbent (soil) used (kg). The percentage of Zn adsorbed or desorbed by the soil was determined from the difference between the initial and equilibrium concentrations for adsorbed Zn and the ratio between desorbed to initial Zn for desorbed Zn [27]:

(2) $$\%\;\mathrm{Adsorption}=\frac{\left(\mathrm{Co}-\mathrm{Ce}\right)}{\mathrm{Co}}*100\%$$

(3) $$\%\;\mathrm{Desorption}=\frac{\mathrm{Cde}}{\mathrm{Co}-\mathrm{Ce}}*100\%$$

where Co, Ce and Cde (mg L^−1^) are the initial, equilibrium and desorbed Zn concentrations in the soil solution, respectively.

### 2.4. Zn Analysis in the Soil Solutions

Portable X-ray fluorescence (pXRF, Tracer 5i, Bruker) was used to measure the amount of Zn in the adsorption and desorption extracts. For this, the pXRF was set to spectrometer mode, selecting the precious metals calibration, configuring the settings to voltage 40 KV and current 40 µA, and 90 s scanning time with Ti/AL filters. First, the equipment was calibrated with the Ag-925 (sterling silver metal for calibrating the Tracer 5i) and the average of fifteen readings was within the range set by the laboratory (8.010–8.323 for Cu and 91.677–91.990 for Ag). Regression analysis between the concentrations of the standard stock solutions (0, 2, 5, 10, 15 and 30 mg L^−1^) and the pXRF readings in pulses gave an r^2^ of 0.99.

### 2.5. Langmuir and Freundlich Isotherm Models

The adsorption and desorption data were fitted to the two most commonly used isotherms in soils. The linear form of the Langmuir isotherm [28] is represented by the following equation:

Langmuir adsorption

(4) $$\frac{\mathrm{Ce}}{\mathrm{Qe}}=\frac{1}{\mathrm{Kb}}+\frac{\mathrm{Ce}}{b}$$

Langmuir desorption

(5) $$\frac{\mathrm{Ce}}{\mathrm{Qde}}=\frac{1}{\mathrm{Kb}}+\frac{\mathrm{Ce}}{b}$$

where Ce (mg L^−1^) is the equilibrium concentration, Qe and Qde (mg kg^−1^) are the amount of adsorbate adsorbed and desorbed per unit mass of adsorbent, and b and K are the Langmuir constants related to adsorption capacity and rate of adsorption and desorption, respectively. The essential characteristics of Langmuir can be expressed by a dimensionless constant called separation factor or equilibrium parameters, R_L_, defined as:

(6) $$\mathrm{RL}=\frac{1}{1+\mathrm{KCo}}$$

where K is the Langmuir constant and Co (mg L^−1^) is the initial Zn concentration. The value of R_L_ indicates the type of isotherm to be either unfavorable (>1), linear (R_L_ = 1), favorable (0 < R_L_ < 1) or reversible (R_L_ = 0).

The linear form of the Freundlich equation [29] is:

Freundlich adsorption

(7) $$\log^{\mathrm{Qe}}=\log^{\mathrm{Kf}}+\frac{1}{n}\log^{\mathrm{Ce}}$$

Freundlich desorption

(8) $$\log^{\mathrm{Qde}}=\log^{\mathrm{Kf}}+\frac{1}{n}\log^{\mathrm{Ce}}$$

where Qe and Qde (mg kg^−1^) are the amount of adsorbed and desorbed at equilibrium and Ce (mg L^−1^) is the equilibrium concentration; Kf and n are Freundlich constants, where n gives an indication of how favorable the adsorption process is; Kf is the adsorption capacity of the adsorbent.

### 2.6. Statistics and Modelling

Multiple linear regression models were developed for adsorption and desorption trends by including those independent variables pH, SOC, eCEC and clay that are known to significantly affect these processes; by including all soil parameters and eliminating those which were not significant through backward elimination; forward selection, forcing the model to have pH, eCEC and SOC in and backwards selection, but first removing high Variance Inflation Factor (VIFs), respectively. The aim was to determine the most explanatory factors affecting Zn adsorption/desorption, and to search for new important factors. Models were fitted in the R statistical environment v. 3.6.2.

## 3. Results

### 3.1. General Soil Physico-Chemical Properties

Generally, the study sites were characterized by increasing pH and decreasing soil organic carbon and total soil N towards lower landscape positions except in the field planted with teff at Aba Gerima (Table 1). No consistent trend with landscape positions could be detected for Olsen P or any of the total elements determined. These soils are classified as strongly to slightly acidic at Aba Gerima teff planted fields and Debre Mewi, strongly to moderately acidic at Aba Gerima maize planted fields while Markuma is characterized by strongly acidic soils [30]. Soil organic carbon (SOC in %) contents of these soils are classified by the same author as low (0.5–1.5%) except at Markuma, which has medium (1.5–3.0%) SOC concentrations. Total nitrogen concentrations (%) are rated as low to moderate for all except for moderate values at Markuma [30]. Available Olsen P concentrations (mg kg^−1^) are generally classified as low [31] and total P (mg kg^−1^) ranges from low to medium [32]. The total concentration of all the secondary macronutrients (Mg and S) fall into medium classes whilst calcium was found to be low at Markuma, medium at Aba Gerima and high at Debre Mewi [32]. With the exception of total Fe and Mn concentrations, which are very high, all the other micronutrients determined (Cu, Mo and Zn) fall in the medium class [32].

Table 2 shows the ammonium oxalate extracts, exchangeable cations, eCEC and soil texture for each site and landscape positions. Generally, ammonium oxalate extractable elements were highly variable and no consistent trend with landscape position could be detected for Al, Fe, Mn and P. Exchangeable cations decreased in the sequence Ca > Mg > K > Na and can be characterized as high for Ca, very low to medium for K, medium to high for Mg and very low for Na [32,33]. The eCEC varied and can be considered medium [32,33], but was generally low at Markuma. Exchangeable cations and eCEC indicate a base saturation between 76% and 91% which corresponds well with the soil pH values in Table 1. The soil texture at all sites ranges from clay-loam to clay, with clay contents between 38% to 70%, and sand contents between 13% to 36%. Again, none of these soil characteristics indicated any clear trend corresponding with the landscape position except texture which usually showed increasingly finer texture (more clay) from the top to the bottom (except for the Aba Gerima fields planted with maize).

### 3.2. Effect of Stock Solution on Equilibrium, Adsorbed and Desorbed Zn

**Figure 2:**
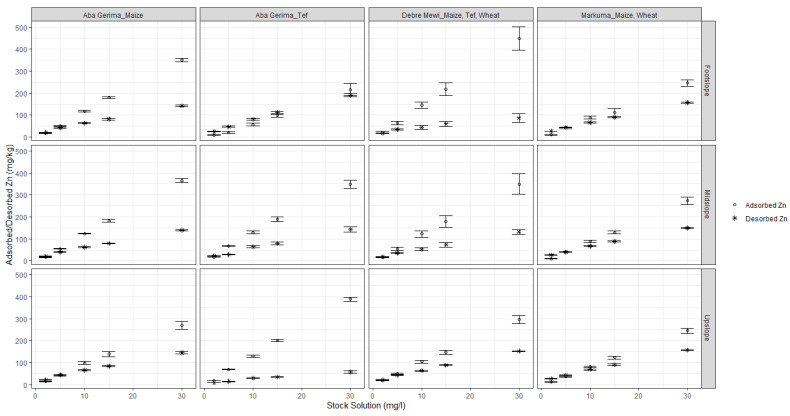
Stock solution concentration on adsorbed and desorbed Zn along the landscape positions.

Regardless of the rate of adsorption, the amount of Zn adsorbed (mg kg^−1^) on the soil particles increased with increasing added Zn concentrations for all sites and landscape positions (Figure 2). The variation in the ranges of Zn adsorption at the different sites could be due to differences in soil characteristics such as pH, clay and soil organic carbon content or CEC. The subsequent Zn desorption also followed similar trends at all sites; the desorbed amount increased with increasing concentration of the previously used adsorption solution, but relatively smaller amounts of Zn desorbed than adsorbed (Figure 2).

**Figure 3:**
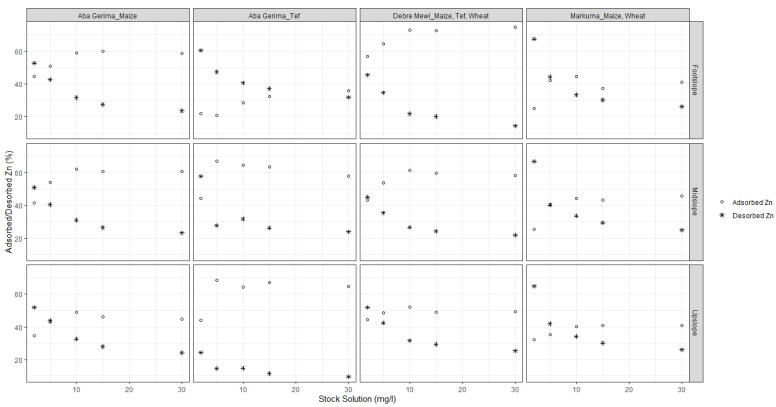
Percentage of adsorbed and desorbed Zn along the landscape positions.

Generally, the percentage of adsorbed Zn increased with increasing initial Zn concentrations at all sites (Figure 3). However, the adsorption rate reached a maximum with stock solutions of 5 mg Zn L^−1^ at for Aba Gerima fields planted with tef while it needed 10 mg Zn L^−1^ for this at Aba Gerima fields planted with maize and at Debre Mewi, except for the footslope at all sites which did not reach a plateau. However in Markuma, the percentage and rate of Zn adsorption kept increasing with increasing initial Zn concentrations in all landscape positions (Figure 3) because most of the soil parameters were similar across the topo sequence (Table 1 and Table 2).

The separation factor R_L_ ranges between 0 and 1 (Table 3) for all soils which indicates that the situation is favorable for reversible adsorption and desorption processes; a R_L_ > 1 means that ad/des is not strong whereas values close to 0 indicate non-reversible processes. The separation factors generally decrease with increasing initial Zn concentrations for all locations and landscape positions. However, at the highest concentration of the stock solution (30 mg L^−1^) the RL factor approaches 0.25 which might indicate a declining rate of desorption for all landscape positions (Figure 3).

In contrast to adsorption, the percentage of desorbed Zn decreases with increasing initial Zn regardless of the location and landscape position (Figure 3). Separation factors followed similar trends to that of adsorption and decreased with increasing initial Zn concentrations.

### 3.3. Comparing the Adsorption-Desorption Results with the Langmuir and Freundlich Isotherms

**Figure 4:**
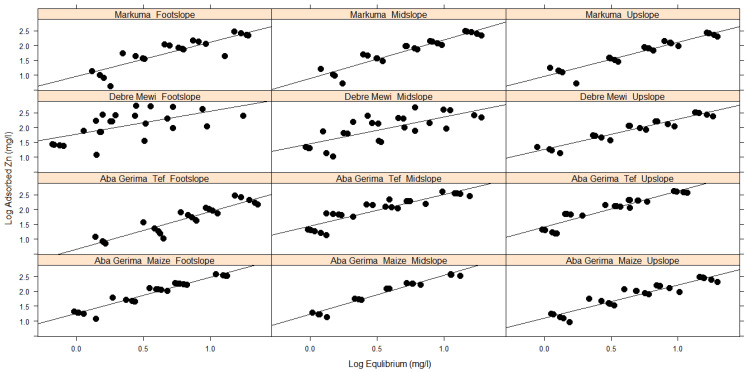
Adsorption data fitted to the Freundlich isotherm.

**Figure 5:**
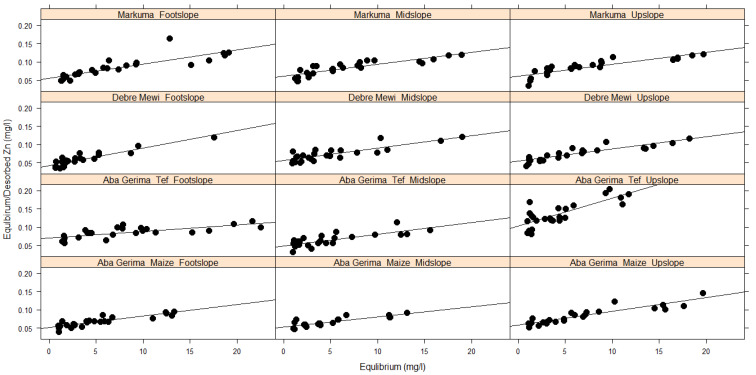
Desorption data fitted to the Langmuir isotherm.

**Figure 6:**
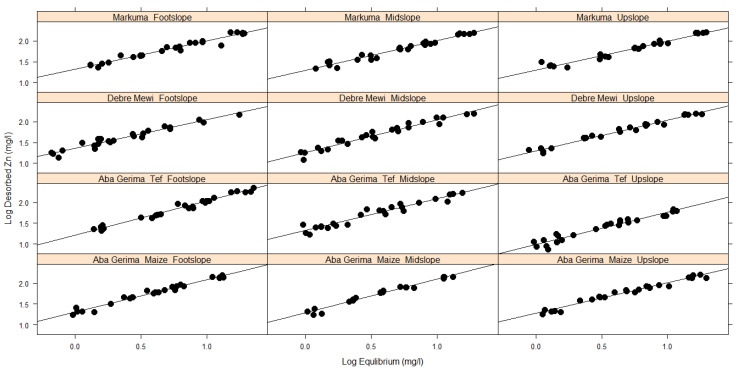
Desorption data fitted to the Freundlich isotherm.

The Langmuir and Freundlich isotherm coefficients for adsorption and desorption, and the respective functions are presented in Table 4 and Table 5 and Figure 4, Figure 5 and Figure 6, respectively. Both Langmuir and Freundlich isotherms, assume linearity in their equations for their respective variables. Accordingly, the Langmuir isotherm assumes linearity when equilibrium Zn vs. the ratio between equilibrium to adsorbed Zn is plotted while the Freundlich isotherm assumes the same for the log equilibrium vs. log adsorbed. The same assumptions are valid for desorption.

Freundlich isotherms were found to fit well for the observed adsorption (Figure 4) and desorption process (Figure 6) for all locations and landscape positions, and similar results were found in [8]. In contrast, Langmuir isotherms described only the desorption processes well (Table 4, Figure 5). These results were confirmed by good relationships (r^2^) between log equilibrium vs. log of adsorbed, log equilibrium vs. log of desorbed and equilibrium vs. ratio of equilibrium to desorbed, respectively (Table 5). Unlike the Freundlich isotherm, which had regression coefficients ≥0.87 for adsorption and desorption across all sites and landscape positions, the Langmuir isotherm achieved variable regressions of between 0.70–0.93 for desorption and between 0.12 and 0.53 for adsorption (Figure 5, Table 4).

### 3.4. Empirical Models with Soil Parameters

Soil factors play different roles with different magnitudes in any dynamic soil nutrient processes. Accordingly, not all soil factors are equally important and do affect the adsorption and desorption processes, and only some will have significant affects [8]. Therefore, we decided to develop functional models with the most relevant soil factors, both from the literature and the current experiments, in order to describe the observed adsorption and desorption processes. Nevertheless, an attempt was made to develop different models including other known factors affecting Zn adsorption/desorption as well as to search for new important factors. However, all models including more factors than pH, SOC and eCEC provided no substantial improvement and tended to overfit the functions for adsorption and desorption. The selected models below conform to statistical assumptions of normality and residual plots and were fitted in the R statistical environment v. 3.6.1. The resulting multiple regression model for adsorption and desorption were:

Multiple regression model for adsorption

(9) $$\mathrm{Adsorption}=-0.92+0.26\mathrm{pH}+0.03\mathrm{SOC};\;\mathrm{adjusted}\;r^{2}=0.90$$

(10) $$\mathrm{Adsorption}=-0.57+0.17\mathrm{pH}+0.04\mathrm{SOC}+0.006\mathrm{eCEC};\;\mathrm{adjusted}\;r^{2}=0.92$$

Multiple regression model for desorption

(11) $$\mathrm{Desorption}=0.89-0.11\mathrm{pH}-0.03\mathrm{SOC};\;\mathrm{adjusted}\;r^{2}=0.70$$

(12) $$\mathrm{Desorption}=0.89-0.11\mathrm{pH}-0.03\mathrm{SOC}-0.00005\mathrm{eCEC};\;\mathrm{adjusted}\;r^{2}=0.69$$

In general, the models were better in predicting adsorption as compared with desorption, and the use of more model parameters improved the prediction for desorption (but had the risk of overfitting). In these models, including eCEC improved the adjusted r^2^ for adsorption very little and gave no improvement for desorption. This implies that in the studied soil, pH and SOC drive the adsorption-desorption process and help to determine the potentially available soil Zn for plant uptakes. These models help to understand and potentially estimate the amount of Zn adsorbed in the soil and desorbed from the soil and, therefore, the plant availability of Zn in these soils. They can also help to guide fertilizer recommendation schemes to improve crop Zn use efficiency as the soil pH and soil organic carbon were found to be influential soil parameters.

## 4. Discussion

### 4.1. Amount of Adsorbed and Desorbed Zn with Landscape Positions

The amount of adsorbed Zn (mg kg^−1^) increased with increasing initial Zn concentrations of the stock solutions, and the amount of Zn desorption increased with increasing amounts of adsorbed Zn (Figure 2). This was as expected and due to the increasing mass transfer driving force from high concentrations (high stock solution Zn concentration or soil with high adsorbed Zn) to low concentrations (low Zn adsorption on the soil or the zero Zn blank solution), resulting in adsorption and desorption processes moving towards an equilibrium. Similar research findings have been reported on many different soils of Tigray [8]. However, the strength of the reactions differed considerably for the different soils.

In all landscape positions, an increase in the adsorption (Figure 2) of Zn was highly associated with an increase of clay content, soil pH, and eCEC of these soils (Table 1 and Table 2). Along the landscape positions from upslope to footslope at Debre Mewi (pH, clay, eCEC) and Aba Gerima fields planted with maize (eCEC), and from footslope to upslope for Aba Gerima fields planted with teff (pH, eCEC), these soil parameters increased, leading to greater adsorption. However, no clear differences were observed among the different landscape positions at Markuma and this was probably because these soil parameters did not differ substantially along the landscape positions. The effects of these soil parameters in the adsorption of Zn has been well studied by several authors. Studies found out that increasing soil pH, soil organic carbon and eCEC significantly increase the amount of adsorbed Zn in soils [8,34,35,36].

Low soil pH in the upslope of Debre Mewi and Aba Gerima fields planted with maize and the footslope of Aba Gerima fields planted with teff could be a stronger driving factor for low adsorption rather than landscape positions. These low pH soils were associated with corresponding low eCEC, hence adsorption is low and Zn is more freely available and can be found in the soil solution [15,16]. In contrast, higher soil pH, usually accompanied by higher eCEC increases adsorption [17]. Except at Debre Mewi, no consistent trends were observed with these soil characteristics and clay content (Table 1 and Table 2). However, increasing clay content increased Zn adsorption, and consequently the activity of Zn in the soil solution decreased with increasing clay content [15]. Furthermore, it seemed that a decrease or increase in soil organic carbon did not influence the adsorption patterns on these locations, possibly because the differences in soil organic carbon were too small and crop management practices are relatively similar within the location. This study aligns with several others that show the activity of Zn in the soil solution increases with decreasing soil pH and decreases with increasing the content of organic carbon and clay particles through adsorption [15,16,17].

Desorption is the opposite of adsorption; as adsorption increases, desorption decreases and vice versa. Desorption continually decreased from upslope to footslope in Aba Gerima fields planted with maize and Debre Mewi while in increased at Aba Gerima fields planted with teff (Figure 2). However at Markuma, desorption was relatively uniform across the landscape positions, most likely because most of the soil parameters such as soil pH, eCEC and even the total Zn content of these soils were similar (Table 1). It has been found that SOC, CEC, and soil pH are the most important factors controlling Zn desorption while calcium carbonate equivalent and clay content were not [37]. In addition, these authors found that soil pH had a negative relationship with Zn desorption. The multiple regression models developed for desorption align with these findings (Equations (3) and (4)).

### 4.2. Langmuir and Freundlich Isotherms

Adsorption and desorption isotherms can be used to describe the equilibrium relationship between the amounts of adsorbed and dissolved species at a given temperature considering the intensity, quantity and capacity factors, which are important for predicting the amount of soil nutrient required for maximum plant growth. In the Langmuir model it is assumed that even at maximum adsorption capacity, there is only a monomolecular layer on the surface. This means that there is no stacking of adsorbed molecules. The Freundlich model does not have this restriction and stacked cation layers are possible. Both models were applied in a number of studies investigating Zn availability in soils and found that soils with divergent characteristics showed good fit to either Langmuir or the Freundlich isotherms [38,39,40]. On calcareous Vertisols soils from Jordan [41] found that both fitted to the soil studied but Freundlich resulted in better fits as compared with Langmuir. Failure of Zn adsorption data to conform to the linear Langmuir equation has been attributed to the existence of more than one type of Zn adsorbing sites, such as occur on different types of clay. In Ethiopia, some of the soil data could not be described with the Langmuir isotherm [8]. The findings by [17] also showed poor fits of soil characteristics to Langmuir adsorption isotherms. The same author reported that the reason for the poor fit was unclear although even though low realistic Zn additions were used for their study. Similarly, the Zn additions in the current study were low representing the low soil Zn status of agricultural soils in Ethiopia. And as described by [17], the Langmuir isotherm did also not fit well to the soils we analyzed, possibly because this isotherm assumes a linear relationship between adsorbed vs. adsorbed/equilibrium Zn variables (Table 4). However, desorption conformed to the Langmuir isotherm (Table 4, Figure 4). In contrast to these results, [42] observed Langmuir isotherm best fits to addition of high Zn concentrations.

The Freundlich isotherm fitted very well to the observed Zn adsorption (Table 5, Figure 4) and desorption (Table 5, Figure 6) for the soils investigated. Similar findings have been reported by [38,39]. Because the Freundlich isotherm is applicable to adsorption and desorption processes that occur on heterogeneous surfaces [43], the good fit of this isotherm indicates that the soil characteristics such as soil pH, eCEC and clay content do vary with landscape positions and locations, significantly affecting the amount of adsorbed and desorbed Zn in the studied soils. The higher r^2^ values for both adsorption and desorption suggested that the Freundlich isotherm is the better model for soils in the studied regions of Ethiopia.

### 4.3. Soil Factors Driving These Processes

Soil pH has been identified in many studies as one of the main factors affecting Zn mobility and sorption in soils [8,15,16]. Zn becomes more soluble as soil pH decreases, it is more mobile and increasingly available in low pH environments, especially below pH 5.0 [44]. As the soil pH at Markuma is classified as strongly acidic (below 4.9 and almost the same for all landscape positions, see Table 1), the rate of adsorption (Figure 3) is very low compared with the moderate Aba Gerima fields planted with Maize, and the less acidic soils at Aba Gerima fields planted with teff and Debre Mewi with soil pH values of 5.5, 6.0 and 6.2, respectively (Table 1, Figure 2).

Absorption and adsorption are two properties related to the surface area of clay minerals. Therefore, the bioavailability of trace elements, including Zn, decreases generally with the clay mineral content in soils [44,45]. Zn may even be irreversibly fixed by clay through isomorphous substitutions or solid-state diffusion into the crystal structure of layered silicates. However, although the soil texture at all the studied sites is classified as clay, the actual clay percentage varied considerably. The amount of clay in Markuma (39–44) was low compared with the other sites which is probably another reason for the low adsorption of Zn at this location. In contrast, high adsorption at Debre Mewi (50–70% clay) is related to the high amount of clay in the soils there, while Aba Gerima fields planted with Maize (47–37% clay) and teff (38–50% clay) had moderate clay content, contributing to the modest Zn adsorption and desorption (Table 2).

In addition, eCEC seems to affect the adsorption of Zn in the studied soils. Ref. [8] found that in his studies of many soils from Tigray region, this was one of the main soil factors affecting adsorption and fitting to the different isotherms. At Debre Mewi (17.0–37.3 cMole kg^−1^) and Aba Gerima fields planted with Maize (13.6–25.9 cMole kg^−1^), the eCEC increased with landscape position which was associated with increasing adsorption and decreasing desorption at these locations. In contrast, at Aba Gerima fields planted with teff (29.8–12.4 cMole kg^−1^) decreasing eCEC values reduced the adsorption and promoted the desorption process. The eCEC values for the Markuma site were similar across landscape positions (12.7–12.5 cMole kg^−1^) and hence the adsorption and desorption processes were similar. eCEC is of course affected by pH, clay content, clay mineralogy and soil organic carbon content, and all these factors interact to produce the observed effects.

### 4.4. Implications for Zn in the Soil and Potential Availability for Crop Uptake

Generally, as soil pH changes across positions in the landscape, the solubility of native soil Zn differs, and adsorption increases with increasing soil pH and vice versa. For example, in the tef planted field at Aba Gerima, soil pH decreased from upslope to footslope while at Debre Mewi it increased with landscape position from upslope to footslope (Table 1). Therefore, in landscape positions with low soil pH, the native soil Zn solubility increased and this, coupled with low adsorption (Equation (1)) and relatively high desorption (Equation (2)), suggests that the application of Zn fertilizers can potentially improve net available soil Zn levels and hence enhance plant Zn uptake.

Soil factors such as soil pH and organic carbon in the studied soils do vary along landscape positions and play a vital role in determining the availability of Zn in the soil by affecting the solubility of native soil Zn reserves and/or added Zn from applied fertilizers through adsorption-desorption process. This will, in turn, determine the net soil Zn potentially available for the plant uptake and improve efficiency. Hence, using important soil factors helps to estimate the amount of Zn in the soil that could be available for crop uptake and would be useful for refining fertilizer recommendation schemes or for suggesting the introduction of Zn uptake efficient crops.

## 5. Conclusions

We conducted this study with the aim of better understanding the soil Zn characteristics along the different landscape positions in order to improve crop uptake through adsorption-desorption studies in Ethiopia. For this objective, we analyzed how well adsorption-desorption data fitted to the most common isotherms and identified the influential soil factors affecting the potentially available soil Zn for uptake by plants.

In general, adsorption fitted to the Freundlich isotherm only while desorption fitted both isotherms. Soil parameters such as pH and SOC were identified as the most factors governing the adsorption and desorption processes and we determined the potential available net soil Zn at different locations. From this study it can be concluded that the most probable reasons for the widespread Zn deficiency in the study is the high rate of Zn adsorption with little desorption. Hence, in areas where the soil has high adsorption capacity, high application rates of Zn fertilizer are needed while soils with low adsorption will need lower rates of Zn fertilizers, which would minimize expense and accumulation of Zn. The models will help to quantify the amount of potentially available soil Zn for crop uptake and can be used to devise stratified Zn fertilizer recommendations for these sites and different landscape positions.

Further studies linking the net potentially available Zn in the soil with plant uptake are needed to better understand uptake efficiencies of different crops and factors affecting plant uptake of Zn from the soil. This will help to improve our understandings on Zn uptake efficiencies on highly adsorptive soils and help in making a decision to select crops which are efficient in the different landscape positions and locations.

## Figures and Tables

**Table 1:** Soil pH, Total Nitrogen, Soil Organic C, Olsen P, and total Ca, Cu, Fe, Mg, Mn, Mo, P, S and Zn. Shown are average values from 5 fields for sites in the same landscape position.

Location_Crop *	Landscape Position	pH	Total N (%)	SOC (%)	Olsen P (mg kg^−1^)		Total Concentrations (mg kg^−1^)								
						Ca	Cu	Fe	K	Mg	Mn	Mo	P	S	Zn
Aba Gerima_T	Upslope	6.0	0.11	1.34	4.2	4470	106	122,623	1514	9469	1815	0.03	289	140	94
	Midslope	5.8	0.08	0.94	3.2	3603	140	125,029	1509	6358	2140	0.02	156	88	114
	Footslope	4.9	0.13	1.41	5.4	1185	78	120,950	2131	3081	1675	0.08	504	180	96
Aba Gerima_M	Upslope	5.2	0.14	1.53	4.8	1769	74	116,409	2220	3276	1513	0.10	605	213	98
	Midslope	5.6	0.12	1.38	3.6	3952	64	115,441	1446	7905	1600	0.03	402	171	97
	Footslope	5.5	0.11	1.31	4.9	3564	69	117,034	1307	7327	1808	0.03	395	144	103
Debre Mewi_MTW	Upslope	5.1	0.17	1.90	5.0	2178	97	112,587	3012	3158	2328	0.20	552	239	101
	Midslope	5.6	0.12	1.37	3.2	3607	64	106,311	2708	4331	1918	0.09	355	148	91
	Footslope	6.2	0.12	1.51	3.3	6190	60	106,482	3084	5334	1991	0.08	233	133	99
Markuma_MW	Upslope	4.8	0.18	2.44	3.9	805	65	102,562	2770	2281	1890	0.48	532	254	55
	Midslope	4.9	0.17	2.27	2.5	909	66	103,972	2967	2287	1800	0.33	499	253	54
	Footslope	4.9	0.15	2.09	2.0	764	70	105,920	2905	2318	1761	0.27	460	231	59
LSD		0.37	0.04	0.47	1.6	1378	36	9987	597	1842	418	0.11	162	61	14
DF		46	46	46	46	46	46	46	46	46	46	46	46	46	46

* Crops grown at the sites were tef (T), maize (M) and wheat (W). LSD is the average Least Significant Differences while DF is degree of freedom.

**Table 2:** Ammonium-Oxalate extractable (Al, Fe, Mn and P), exchangeable cations (Ca, K, Mg and Na), effective cation exchange capacity (eCEC), base saturation and soil texture. Shown are average values from 5 fields per each site in the same landscape position.

Location_Crop *	Landscape Position	AmmOx (mg kg^−1^)				Exchangeable Cations (cMolc kg^−1^)				eCEC (cMolc kg^−1^)	Base Saturation	Soil Texture (%)			Texture Class, USDA
		Al	Fe	Mn	P	Ca	K	Mg	Na		%	Sand	Silt	Clay	
Aba Gerima_T	Upslope	6882	14,172	1218	232	16.7	0.3	10.0	0.10	29.8	91	34	28	38	CL
	Midslope	4358	11,411	1514	138	15.2	0.1	9.1	0.10	27.8	89	36	28	36	CL
	Footslope	5031	12,348	1127	168	6.3	0.1	3.0	0.04	12.4	76	25	25	50	C
Aba Gerima_M	Upslope	4636	13,103	1036	180	8.6	0.3	3.2	0.05	13.6	89	25	28	47	C
	Midslope	5473	12,957	1085	170	15.4	0.3	10.2	0.06	28.3	91	29	28	43	C
	Footslope	5139	14,245	1288	176	13.8	0.1	9.0	0.07	25.9	89	31	32	37	CL
Debre Mewi_MTW	Upslope	3940	10,347	1894	153	9.9	0.3	4.4	0.05	17.0	86	23	27	50	C
	Midslope	3706	9012	1537	94	16.2	0.3	6.7	0.07	25.8	90	21	22	57	C
	Footslope	3351	9759	1657	82	25.7	0.4	7.8	0.06	37.3	91	13	17	70	C
Markuma_MW	Upslope	4767	12,527	1396	162	7.7	0.1	2.8	0.03	12.7	84	29	32	39	CL
	Midslope	4057	10,492	1284	136	7.9	0.2	2.9	0.02	13.0	85	28	30	42	C
	Footslope	4422	10,602	1242	142	7.2	0.2	2.8	0.02	12.5	82	27	29	44	C
LSD		784	2781	392	83	5	0.2	2.6	0.02	7.5	6	5	5	8	
DF		46	46	46	46	46	46	46	46	46	46	46	46	46	

* Crops grown at the sites were tef (T), maize (M) and wheat (W); CL = Clay Loam, C = Clay; LSD is the average Least Significant Differences while DF is degree of freedom; AmmOx- Ammonium-Oxalate extractable Aluminium (Al), Iron (Fe), Manganese (Mn) and Phosphorus (P).

**Table 3:** Summary of the RL factor for adsorption and desorption.

Landscape Position	Co (mg L^−1^)	Aba Gerima_Tef		Aba Gerima_Maize		Debre Mewi_Maize, Tef, Wheat		Markuma_Maize, Wheat	
		RL_Ad	RL_De	RL_Ad	RL_De	RL_Ad	RL_De	RL_Ad	RL_De
Upslope	2	0.92	0.84	0.87	0.89	0.90	0.90	0.84	0.89
	5	0.83	0.67	0.73	0.77	0.78	0.78	0.67	0.77
	10	0.71	0.50	0.57	0.63	0.64	0.64	0.51	0.62
	15	0.62	0.40	0.47	0.53	0.54	0.55	0.41	0.52
	30	0.45	0.25	0.31	0.36	0.37	0.38	0.26	0.35
Midslope	2	0.93	0.91	0.90	0.90	0.91	0.89	0.82	0.89
	5	0.84	0.80	0.79	0.79	0.80	0.77	0.65	0.77
	10	0.72	0.67	0.65	0.65	0.66	0.62	0.48	0.62
	15	0.63	0.58	0.55	0.56	0.57	0.52	0.38	0.52
	30	0.46	0.41	0.38	0.39	0.40	0.35	0.24	0.35
Footslope	2	0.73	0.88	0.90	0.91	0.95	0.93	0.83	0.90
	5	0.52	0.74	0.79	0.80	0.89	0.84	0.66	0.77
	10	0.35	0.58	0.66	0.66	0.80	0.73	0.49	0.63
	15	0.26	0.48	0.56	0.57	0.73	0.64	0.39	0.53
	30	0.15	0.32	0.39	0.40	0.57	0.44	0.24	0.36
LSD		0.10	0.11	0.10	0.11	0.09	0.11	0.12	0.11
DF		72	72	67	67	67	67	72	72

RL-Ad and RL_De refers to separation factor for adsorption and desorption, respectively; LSD is the average Least Significant Differences while DF is degree of freedom.

**Table 4:** Langmuir coefficients for adsorption and desorption isotherms.

Location_Crop *	Landscape Position	Langmuir Isotherm Coefficients					
		Adsorption			Desorption		
		b	K	r^2^	b	K	r^2^
Aba Gerima_T	Upslope	−646	−0.04	0.16	152	0.09	0.75
	Midslope	−890	−0.02	0.17	365	0.07	0.72
	Footslope	−452	−0.03	0.41	753	0.02	0.55
Aba Gerima_M	Upslope	3020	−0.02	0.18	305	0.06	0.93
	Midslope	−420	−0.05	0.35	377	0.05	0.80
	Footslope	−671	−0.03	0.36	334	0.06	0.85
Debre Mewi_MTW	Upslope	−27554	−0.01	0.12	329	0.06	0.86
	Midslope	−1061	−0.05	0.20	45	0.04	0.72
	Footslope	−654	−0.18	0.53	165	0.21	0.70
Markuma_MW	Upslope	−877	−0.02	0.30	313	0.05	0.80
	Midslope	−324	−0.04	0.44	333	0.05	0.81
	Footslope	−211	−0.02	0.21	289	0.06	0.83
LSD		4716	0.02		90	0.02	
DF		285	285		285	285	

* Crops grown at the sites were tef (T), maize (M) and wheat (W).

**Table 5:** Freundlich coefficients for adsorption and desorption isotherms.

Location_Crop *	Landscape Position	Freundlich Isotherm Coefficients					
		Adsorption			Desorption		
		1/n	Kf	r^2^	1/n	Kf	r^2^
Aba Gerima_T	Upslope	1.24	1.39	0.90	0.78	0.99	0.96
	Midslope	0.91	1.43	0.88	1.33	1.33	0.95
	Footslope	0.79	0.63	0.91	1.21	1.21	0.99
Aba Gerima_M	Upslope	1.17	1.06	0.97	0.75	1.27	0.99
	Midslope	0.76	1.24	0.96	1.21	1.29	0.98
	Footslope	0.80	1.23	0.97	1.28	1.31	0.98
Debre Mewi_MTW	Upslope	0.93	1.23	0.99	1.34	1.29	0.99
	Midslope	0.80	1.27	0.95	1.20	1.24	0.98
	Footslope	0.64	1.68	0.94	1.40	1.36	0.92
Markuma_MW	Upslope	0.87	0.95	0.97	1.44	1.30	0.98
	Midslope	0.75	0.86	0.96	1.38	1.28	0.98
	Footslope	0.86	0.92	0.87	1.46	1.32	0.98
LSD		0.09	0.10		0.03	0.03	
DF		285	285		285	285	

* Crops grown at the sites were tef (T), maize (M) and wheat (W).
